# Clonidine for sedation in the critically ill: a systematic review and meta-analysis

**DOI:** 10.1186/s13054-017-1610-8

**Published:** 2017-02-25

**Authors:** Jing Gennie Wang, Emilie Belley-Coté, Lisa Burry, Mark Duffett, Timothy Karachi, Dan Perri, Waleed Alhazzani, Frederick D’Aragon, Hannah Wunsch, Bram Rochwerg

**Affiliations:** 1 Department of Medicine, Faculty of Health Sciences, McMaster University, Hamilton, ON Canada; 2 Department of Health Research Methods, Evidence & Impact, McMaster University, Hamilton, ON Canada; 3 Department of Pharmacy, Mount Sinai Hospital, Toronto, ON Canada; 4 Leslie Dan Faculty of Pharmacy, University of Toronto, Toronto, ON Canada; 5 Department of Pediatrics, McMaster University, Hamilton, ON Canada; 6 Hamilton Health Sciences, Hamilton, ON Canada; 7 St. Joseph’s Healthcare Hamilton, Hamilton, ON Canada; 8 Department of Critical Care Medicine, Sunnybrook Health Sciences Centre, Toronto, ON Canada; 9 Department of Anesthesia, University of Toronto, Toronto, ON Canada; 10 Interdepartmental Division of Critical Care Medicine, University of Toronto, Toronto, ON Canada

**Keywords:** Clonidine, Systematic review, Sedation, Delirium, Mechanical ventilation, Weaning

## Abstract

**Background:**

This systematic review and meta-analysis investigates the efficacy and safety of clonidine as a sedative in critically ill patients requiring invasive mechanical ventilation.

**Methods:**

We performed a comprehensive search of MEDLINE, EMBASE, CINAHL and the Cochrane trial registry. We identified RCTs that compared clonidine to any non-clonidine regimen in critically ill patients, excluding neonates, requiring mechanical ventilation. The GRADE method was used to assess certainty of evidence.

**Results:**

We included eight RCTs (*n* = 642 patients). In seven of the trials clonidine was used for adjunctive rather than stand-alone sedation. There was no difference in the duration of mechanical ventilation (mean difference (MD) 0.05 days, 95% confidence interval (CI) = -0.65 to 0.75, *I* ^*2*^ = 86%, moderate certainty), ICU mortality (relative risk (RR) 0.98, 95% CI = 0.51 to 1.90, *I* ^*2*^ = 0%, low certainty), or ICU length of stay (MD 0.04 days, 95% CI = -0.46 to 0.53, *I* ^*2*^ = 16%, moderate certainty), with clonidine. There was a significant reduction in the total dose of narcotics (standard mean difference (SMD) -0.26, 95% CI = -0.50 to -0.02, *I* ^*2*^ = 0%, moderate certainty) with clonidine use. Clonidine was associated with increased incidence of clinically significant hypotension (RR 3.11, 95% CI = 1.64 to 5.87, *I* ^*2*^ = 0%, moderate certainty).

**Conclusions:**

Until further RCTs are performed, data remains insufficient to support the routine use of clonidine as a sedative in the mechanically ventilated population. Clonidine may act as a narcotic-sparing agent, albeit with an increased risk of clinically significant hypotension.

## Background

Critically ill patients requiring invasive mechanical ventilation (IMV) usually require sedation to minimize discomfort, reduce the risks of self-injury and facilitate care [1, 2]. Randomized controlled trials (RCTs) have demonstrated clear benefits of minimizing sedation in this population, such as a reduction in the duration of mechanical ventilation [3, 4], shorter length of stay in the intensive care unit (ICU) [4–6] and improved overall survival [6].

Typical sedatives used in patients requiring IMV include propofol, benzodiazepines and more recently, dexmedetomidine [7]. Although propofol has a rapid onset of action and provides timely recovery after discontinuation, it can cause clinically significant hypotension [8]. Benzodiazepines may increase the risk of ICU-related delirium and cause over-sedation due to drug accumulation, prolonging the duration of IMV [9]. Compared to benzodiazepines, dexmedetomidine reduces the incidence of delirium and the duration of IMV [10–12], but is not widely available due to cost.

Clonidine stimulates pre-synaptic alpha-2 adrenoreceptors within the brainstem, decreasing norepinephrine release while enhancing parasympathetic activity. The sedative, analgesic and anxiolytic effects of clonidine may be due to its effects on the locus coeruleus [13]. Evidence supporting the use of clonidine as a sedative in the critically ill requiring IMV remains scarce. One recent systematic review on the efficacy of alpha-2 agonists for sedation in the pediatric critically ill population included three RCTs using clonidine, but did not pool estimates. They concluded that robust evidence was lacking for the use of clonidine as a sedative in the pediatric critically ill population [14]. A Cochrane meta-analysis that assessed the efficacy of alpha-2 agonists on the quality of sedation in ventilated critically ill patients did not include any studies on clonidine [15]. The 2013 Pain, Agitation, and Delirium guidelines make no recommendation on the use of clonidine [16]. The objective of our systematic review is to summarize the available RCT evidence on the use of clonidine as a sedative in the ICU in order to better inform clinical practice.

## Methods

### Data sources and searches

We performed a comprehensive search of MEDLINE, Excerpta Medica database (EMBASE), Cumulative Index to Nursing and Allied Health Literature (CINAHL), and the Cochrane trial registry from inception until March 2016 (Appendix 1). No date or language restrictions were applied. Two reviewers independently screened all references for inclusion and a third party resolved discrepancies. We identified unpublished and ongoing trials using the World Health Organization International Clinical Trials Registry Platform (WHO ICTRP) and clinicaltrials.gov databases. Conference proceedings for the Society of Critical Care Medicine (SCCM), Canadian Critical Care Society, the European Society of Intensive Care Medicine (ESICM), and the American Thoracic Society (ATS) were screened in duplicate for the last 2 years.

### Study selection

No methodological quality restrictions were imposed. Although non-randomized prospective studies were identified in the initial search, a sufficient number of RCTs were identified such that only RCT data were subsequently analyzed and reported. Eligible studies were RCTs reporting the use of clonidine, either as a primary sedative or adjunctive agent, compared to any non-clonidine sedative regimen, in patients who required IMV. Studies that used clonidine for any indication other than sedation (e.g. opioid withdrawal) were excluded. We excluded studies enrolling only neonates and those in which clonidine was administered by a route other than enteral or intravenous (IV).

We included studies that reported any of our a priori outcomes, namely the duration of mechanical ventilation, duration of non-invasive ventilation (NIV), all-cause mortality, duration of sedative infusion, dose of benzodiazepines or narcotics used during ICU stay, the level of sedation, incidence of withdrawal from other sedatives, incidence of delirium, and ICU and hospital length of stay. Adverse events were also captured, including clinically significant bradycardia and hypotension requiring intervention, clonidine withdrawal symptoms (rebound hypertension), the unplanned removal of support lines and unplanned extubation.

### Data extraction and quality assessment

Data extraction was performed independently and in duplicate using predefined data abstraction forms. A third reviewer resolved disagreements when necessary.

Independently and in duplicate, two reviewers assessed the risk of bias (ROB) for each outcome of individual studies using the Cochrane ROB tool [17]. The ROB was judged to be “low risk,” “high risk” or “unclear risk” within the following domains: sequence generation, allocation sequence concealment, blinding, selective outcome reporting and other bias. We assessed the overall certainty of evidence using the Grading of Recommendations Assessment, Development and Evaluation (GRADE) method [18] for each outcome independently. Disagreements for ROB and GRADE assessments were resolved by discussion and consensus.

### Data analysis

Results are presented as relative risk (RR) with 95% confidence interval (CI) for dichotomous outcomes and as mean difference (MD) or standardized mean difference (SMD) for continuous outcomes with 95% CI. Meta-analyses were conducted on pooled outcomes using Review Manager 5.3. Random effects model analysis was performed for all outcomes and study weights were measured using the inverse variance strategy, in the method of DerSimonian and Laird [19].

Heterogeneity was assessed using the chi-squared test for homogeneity, and the *I* ^*2*^ statistic [20]; *I* ^*2*^ greater than 50% was considered significant heterogeneity. The Egger test was not performed as less than ten trials were identified [21]. We used the GRADEPro guideline development tool to formulate GRADE evidence profiles [22].

### Outcomes

Primary and secondary outcomes of interest were described a priori in a separately published protocol [23]. As per the predefined protocol, outcomes were pooled across studies and described narratively if pooling was not possible. Subgroup and sensitivity analyses were not conducted due to the limited number of trials identified per outcome.

## Results

### Study identification

**Fig. 1:**
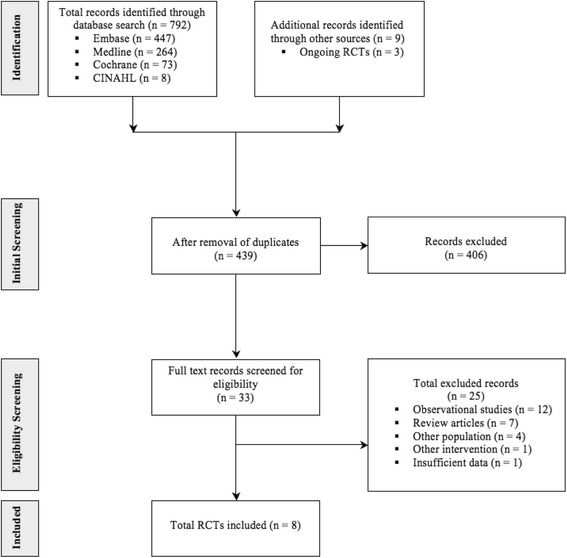
Flow diagram depicting a summary of the search and selection process. *CINAHL* Cumulative Index to Nursing and Allied Health Literature, *EMBASE* Excerpta Medica database, *RCTs* randomized controlled trials

Of an initial 792 citations, 33 underwent full text review. After excluding a further 25 studies, a total of eight RCTs met inclusion criteria [24–31]. In addition, we identified three ongoing RCTs (NCT01139996, NCT02509273, NCT01876355) (Fig. 1).

### Study characteristics

A detailed description of the included trials is presented in Table 1. Four trials enrolled children [24–26, 29] and four enrolled adults [27, 28, 30, 31]. Clonidine was administered intravenously in six trials [24, 26–30] and via the enteral route in two trials [25, 31]. The trials that used intravenous clonidine took place in Brazil, the United Kingdom, Germany, India and Italy [24, 26–30], respectively. The doses of clonidine varied considerably, with enteral clonidine doses ranging from 0.1 to 0.2 mg every 8 hours [31] to 5 μg/kg every 6 hours [25]. The doses for IV continuous clonidine infusions ranged from 0.88 to 3 μg/kg/hour [26–30] and some studies used an initial bolus dose [26, 28, 30]. One study used intermittent IV doses of clonidine at 5 μg/kg every 8 hours [24]. Most trials used clonidine as an adjunctive agent added to an established sedative regimen, generally consisting of a benzodiazepine and/or an opioid [24–26, 29–31]. A single trial used clonidine as a stand-alone agent, compared to dexmedetomidine [27].

**Table 1:** Description of the characteristics of the included randomized controlled trials

Trial	Population	Intervention	Clonidine: adjunctive or stand-alone sedative agent	Comparator(s)	Outcome(s) of interest
Duffett et al., 2014	Mechanically ventilated children 1 month to 18 years old	*n* = 25 Clonidine 5 μg/kg enteral (max 200 μg) q6h	Adjunctive (Midazolam, morphine equivalents)	n = 25 Placebo	Level of sedation (COMFORT or State Behavioural scales), dose of sedative agents (midazolam and morphine equivalents), duration of mechanical ventilation, length of PICU/hospital stay, adverse events
Farasatinasab et a.l, 2015	Mechanically ventilated adults	*n* = 30 Clonidine 0.1–0.2 mg enteral q8h	Adjunctive (Midazolam, morphine equivalents, propofol)	*n* = 25 Placebo	Level of sedation (Ramsay Sedation Score), dose of sedative agents (midazolam and morphine equivalents), total amount of sedation used, adverse events
Hünseler et al., 2014	Mechanically ventilated newborns and children 0 days to 2 years old. The newborn subgroup was excluded in data analysis	*n* = 105 Clonidine 1 μg/kg/hr infusion	Adjunctive (Midazolam, fentanyl, thiopentone prn)	*n* = 114 Placebo	Level of sedation (Hartwig and COMFORT scores), dose of sedative agents (fentanyl and midazolam), duration of mechanical ventilation, length of ICU stay, mortality
Rubino et al., 2009	Mechanically ventilated adults who had undergone surgical correction of acute type A aortic dissection	*n* = 15 Clonidine 0.5 μg/kg intravenous bolus, then 1–2 μg/kg /hr infusion	Adjunctive (Fentanyl, propofol)	*n* = 15 Placebo	Incidence of delirium, severity of delirium (Delirium Detection Score), duration of weaning, length of ICU stay
Spies et al., 1996	Mechanically ventilated post-trauma surgery adults who were alcohol-dependent	*n* = 54 Clonidine 0.3 mg intravenous bolus, then up to 0.88 μg/kg/hr infusion and flunitrazepam 4 mg intravenous bolus, then up to 19 μg/kg/hr infusion	Adjunctive (Flunitrazepam)	(1) *n* = 50 Chlormethiazole 375 mg intravenous bolus, then up to 8.2 mg/kg/hr infusion and haloperidol 20 mg intravenous, then up to 53 μg/kg/hr infusion. (2) *n* = 55 Flunitrazepam 6 mg intravenous bolus, then up to 28 μg/kg/hr infusion and haloperidol 20 mg intravenous bolus, then up to 87 μg/kg/hr infusion	Length of ICU stay, incidence of alcohol withdrawal syndrome (revised clinical institute withdrawal assessment for alcohol scale), adverse events
Srivastava et al., 2014	Mechanically ventilated adults	*n* = 35 Clonidine 1–2 μg/kg/hr infusion	Stand-alone	*n* = 35 Dexmedetomidine 0.7 μg/kg intravenous bolus, then 0.2–0.7 μg/kg/hr infusion	Level of sedation (Ramsay sedation score), dose of sedative agents (diazepam, dexmedetomidine, fentanyl), hemodynamic changes, adverse events
Wolf et al., 2014	Mechanically ventilated children age 30 days to 15 years	*n* = 61 Clonidine 3 μg/kg intravenous bolus, then 0–3 μg /kg/hr infusion	Adjunctive (Midazolam, morphine)	*n* = 59 Midazolam 200 μg/kg intravenous bolus, then 0–20 μg/kg/hr infusion	Level of sedation (COMFORT score), time spent adequately sedated, duration of sedation, adverse events, length of ICU/hospital stay, ICU mortality
Molon et al., 2007	Mechanically ventilated children (age criteria unspecified)	*n* = 31 Clonidine 5 μg/kg intravenous q8h	Adjunctive (Midazolam, morphine)	*n* = 38 Placebo	Dose of sedatives (midazolam and morphine), duration of sedation, incidence of withdrawal syndrome (Finnegan score)

### Risk of bias

ROB was reported using the Cochrane ROB tool for each individual study (Appendix 2) [17]. Overall, two trials were at low ROB [25, 29] and six trials at high ROB [24, 26–28, 30, 31]. Of the high ROB trials, one did not specify blinding details and had a high risk of attrition bias (33% of patients in the clonidine group were lost to follow-up) [31]. Another trial had a high risk of selection bias, as study investigators did not specify whether the envelopes used for randomization were sealed or opaque [30]. One did not blind patients or caregivers and excluded 21 of 180 patients post-randomization [28]. One was an open-label study, with associated risks of performance and detection bias [27]. Four did not describe allocation concealment [24, 26, 28, 31].

### Certainty of evidence

Each outcome was rated on the certainty in effect estimates using the GRADE approach (Table 2).

**Table 2:** The GRADE approach was used to assess the certainty of evidence

Quality assessment							№ of patients		Effect		Quality	Importance
№ of studies	Study design	Risk of bias	Inconsistency	Indirectness	Imprecision	Other considerations	Clonidine	Placebo	Relative (95% CI)	Absolute (95% CI)		
Duration of mechanical ventilation (assessed with: days)												
6	Randomized trials	Not serious^a^	Serious^b^	Not serious	Not serious^c^	None	200	217	-	MD 0.05 days more (0.65 fewer to 0.75 more)	⨁⨁⨁ MODERATE	CRITICAL
ICU mortality												
5	Randomized trials	Serious^d^	Not serious	Not serious	Serious^e^	None	14/164 (8.5%)	23/219 (10.5%)	RR 1.00 (0.50 to 2.02)	0 fewer per 1000(from 53 fewer to 107 more)	⨁⨁ LOW	CRITICAL
Dose of benzodiazepines												
4	Randomized trials	Not serious^f^	Serious^g^	Not serious	Not serious^c^	None	130	134	-	SMD 0.02 SD higher (0.34 lower to 0.39 higher)	⨁⨁⨁ MODERATE	CRITICAL
Dose of narcotics												
4	Randomized trials	Not serious^f^	Not serious	Not serious	Serious^e^	None	130	134	-	SMD 0.26 SD lower (0.5 lower to 0.02 lower)	⨁⨁⨁ MODERATE	CRITICAL
Incidence of withdrawal symptoms from other sedatives												
3	Randomized trials	Serious^i^	Not serious	Not serious	Serious^h^	None	40/120 (33.3%)	49/124 (39.5%)	RR 0.91 (0.67 to 1.23)	36 fewer per 1000 (from 91 more to 130 fewer)	⨁⨁ LOW	IMPORTANT
ICU length of stay												
6	Randomized trials	Serious^j^	Not serious	Not serious	Not serious^c^	None	233	240	-	MD 0.04 days more (0.46 fewer to 0.53 more)	⨁⨁⨁ MODERATE	CRITICAL
Incidence of clinically significant hypotension												
4	Randomized trials	Not serious^k^	Not serious	Not serious	Serious^l^	None	31/178 (17.4%)	12/226 (5.3%)	RR 3.11 (1.64 to 5.87)	112 more per 1000 (from 34 more to 259 more)	⨁⨁⨁ MODERATE	CRITICAL
Incidence of clinically significant bradycardia												
4	Randomized trials	Not serious^k^	Not serious	Not serious	Very serious^e^	None	14/178 (7.9%)	12/226 (5.3%)	RR 1.34 (0.45 to 3.98)	18 more per 1000 (from 29 fewer to 158 more)	⨁⨁ LOW	CRITICAL

*CI* confidence interval, *MD* mean difference, *RR* risk ratio, *SMD* standardized mean difference

^a^Four of six included studies had issues related to high risk of bias. However, excluding high risk of bias studies had no effect on overall pooled estimates

^b^High *I* ^*2*^ value of 86%

^c^Point estimate shows no effect. Confidence intervals do not rule out slight harm or slight benefit, however, neither meet clinical significance threshold

^d^All included studies for this outcome were at high risk of bias

^e^Confidence intervals do not exclude benefit or harm

Confidence intervals do not exclude benefit or harm. Low number of patients and event numbers

Two of three trials had issues related to high risk of bias. However, sensitivity analysis excluding high risk of bias trials did not alter results

High *I* ^*2*^ value of 82%

Confidence intervals do not exclude benefit or harm, with the benefit potentially meeting clinical threshold. Low number of patients

^f^Two of four trials had issues related to high risk of bias. However sensitivity analysis excluding high risk of bias trials did not alter results

^g^High *I* ^*2*^ value of 55%

^h^Confidence intervals do not exclude benefit or harm. Low number of patients

^i^Two of three trials had issues related to high risk of bias. However, sensitivity analysis excluding high risk of bias trials results in a potentially different outcome

^j^Four of six trials had issues related to high risk of bias. However, sensitivity analysis excluding high risk of bias trials results in a potentially different outcome

High *I* ^*2*^ value of 52%

^k^Three of four trials had issues related to high risk of bias. However sensitivity analysis excluding the high risk of bias trials did not alter results

^l^Low number of events

### Pooled outcomes

#### Duration of mechanical ventilation

**Fig. 2:**
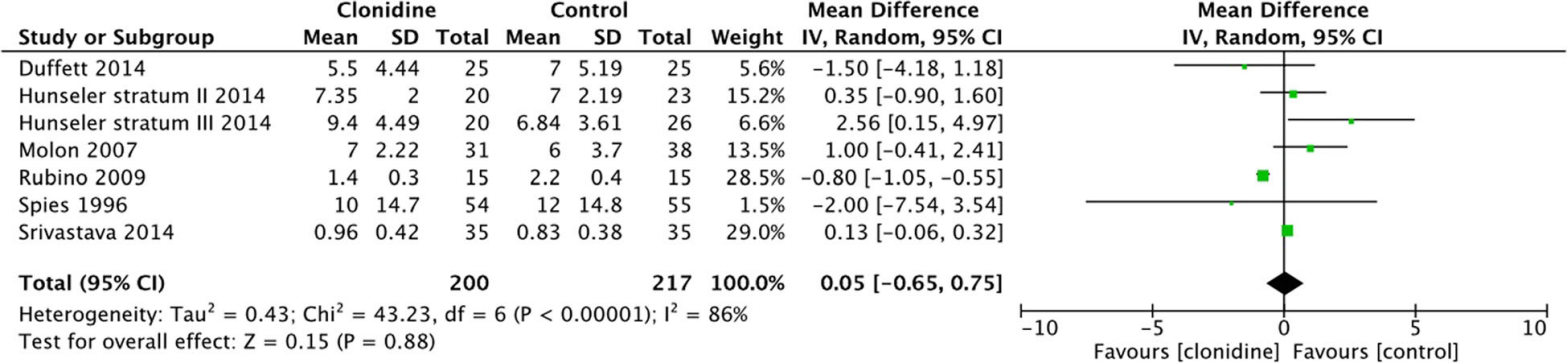
Forest plot comparing the duration of mechanical ventilation between the clonidine group and the non-clonidine group (control). Results are depicted using a random effects model with mean difference and 95% confidence intervals

The duration of mechanical ventilation was similar for patients receiving clonidine and those in the non-clonidine group (six studies, *n* = 417 patients, MD 0.05 days, 95% CI -0.65 to 0.75, *I* ^*2*^ = 86%, moderate certainty) [24, 25, 27–30] (Fig. 2). There was insufficient data to comment on the use and duration of NIV.

#### All cause mortality

There was no difference in ICU mortality (five studies, *n* = 383 patients, RR 0.98, 95% CI = 0.51 to 1.90, *I* ^*2*^ *=* 0%, low certainty) [24, 26–28, 30] (Appendix 3), or hospital mortality (two studies, *n* = 139 patients, RR 0.37, 95% CI = 0.08 to 1.76, *I* ^*2*^ = 0%, moderate certainty) [25, 29] (Appendix 4) between the clonidine and the non-clonidine group.

#### Other sedatives, analgesics and sedation parameters

**Fig. 3:**
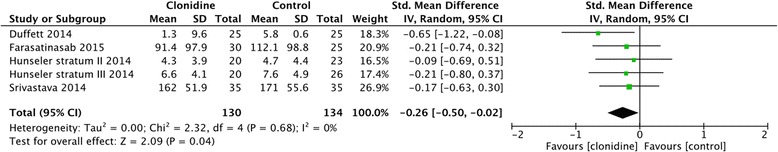
Forest plot comparing the dose of narcotics used between the clonidine group and the non-clonidine group (control). Results are depicted using a random effects model with standard mean difference and 95% confidence intervals

There was no difference in the duration of sedative infusions (three studies, 245 patients, MD -0.28 days, 95% CI = -0.91 to 0.34, *I* ^*2*^ = 82%, low certainty) [25–27] (Appendix 5), or total dose of benzodiazepines (four studies, 264 patients, SMD 0.02, 95% CI = -0.34 to 0.39, *I* ^*2*^ = 55%, moderate certainty) [25, 27, 29, 31] (Appendix 6) between the clonidine and non-clonidine groups. The total dose of narcotics was significantly reduced in the clonidine group compared to the non-clonidine group (four studies, 264 patients, standard mean difference (SMD) -0.26, 95% CI = -0.50 to -0.02, *I* ^*2*^ = 0%, moderate certainty) [25, 27, 29, 31] (Fig. 3).

Four RCTs reported the level of sedation achieved. Two RCTs used a sedation scoring system and reported this as a continuous outcome, which allowed pooling [25, 29]. Analysis showed no difference in the level of sedation achieved in the clonidine compared to the non-clonidine group (two studies, 139 patients, SMD -0.28, 95% CI = -0.61 to 0.06, *I* ^*2*^ = 0%, moderate certainty) [25, 29] (Appendix 7).

#### Withdrawal from other sedatives

Three RCTs reported the incidence of withdrawal from other sedatives [24–26]. This was defined using a withdrawal diagnostic tool, namely the Finnegan score [24], the Withdrawal Assessment Tool 1 [25] and an 11-point assessment for abnormal behaviors [26]. Overall, there was no significant difference in the incidence of withdrawal from other sedatives between groups (three studies, 244 patients, RR 0.91, 95% CI = 0.67 to 1.23, *I* ^*2*^ = 0%, low certainty) [24–26] (Appendix 8). There was insufficient data to comment on the incidence of delirium.

#### ICU and hospital length of stay

There was no difference in the ICU length of stay (six trials, 473 patients, MD 0.04 days, 95% CI = -0.46 to 0.53, *I* ^*2*^ = 16%, moderate certainty) [25–30] (Appendix 9) or hospital length of stay (three studies, 245 patients, MD -0.66, 95% CI = -2.18 to 0.87, *I* ^*2*^ = 52%, very low certainty) [25–27] (Appendix 10) between the clonidine and non-clonidine groups.

#### Adverse events

**Fig. 4:**
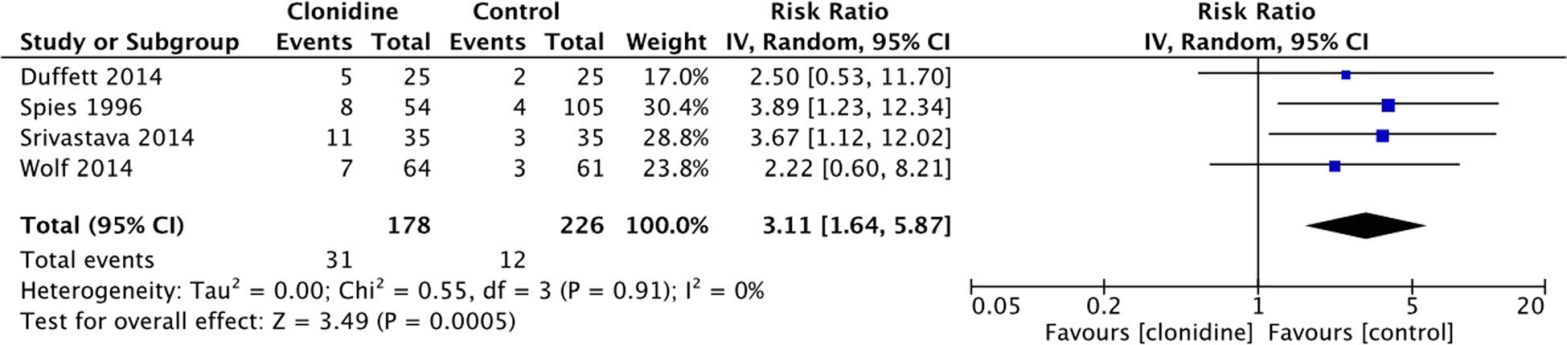
Forest plot comparing the incidence of clinically significant hypotension requiring intervention between the clonidine group and the non-clonidine group (control). Results are depicted using a random effects model with relative risk ratio and 95% confidence intervals

An increased incidence of clinically significant hypotension requiring intervention was evident in the clonidine compared to the non-clonidine group (four studies, 404 patients, RR 3.11, 95% CI = 1.64 to 5.87, *I* ^*2*^ = 0%, moderate certainty) [25–28] (Fig. 4). Two studies defined clinically significant hypotension as any decrease in blood pressure requiring intervention, such as holding or lowering the dose of clonidine, or requiring administration of intravenous fluids [25, 26]. One study used a systolic blood pressure cutoff of 80 mmHg, diastolic blood pressure cutoff of 50 mmHg, or change in baseline blood pressure of >30% as criteria for clinically significant hypotension [27]. Another study defined it as a blood pressure <60 mmHg (unclear if this is mean arterial pressure, systolic or diastolic blood pressure), or any hypotension requiring intervention with a vasopressor or inotrope [28].

There was no difference in the incidence of clinically significant bradycardia requiring intervention (four studies, 404 patients, RR 1.34, 95% CI = 0.45 to 3.98, *I* ^*2*^ = 45%, low certainty) [25–28] (Appendix 11) or the incidence of rebound hypertension (two studies, 195 patients, RR 5.37, 95% CI = 0.63 to 45.49, *I* ^*2*^ = 0%, low certainty) [26, 27] (Appendix 12). None of the included studies reported on the incidence of unplanned removal of central IV lines or unplanned extubation.

## Discussion

We found no significant difference in the duration of IMV, ICU mortality, duration of sedation infusion or ICU length of stay between the clonidine and non-clonidine groups. However, a high degree of clinical heterogeneity limits the interpretation of these results. Included studies were heterogeneous with regards to patient age (adults and pediatric patients), and types of patients, including medical, post-operative or mixed groups. Notably, the patients in two of the trials were post-operative and thus mechanically ventilated for only a brief period (mean < 72 hours) [28, 30], making it less likely that these studies would demonstrate significant differences in the duration of IMV. Further, clonidine dosing and route of administration, as well as the comparators used, varied across trials. This may have contributed to a high degree of statistical heterogeneity for some outcomes. Unfortunately, due to the scarcity of evidence, a priori planned subgroup analyses attempting to explain this heterogeneity was not possible.

The level of sedation achieved did not differ significantly between the clonidine and non-clonidine groups. However, pooled analysis was limited as the reporting mechanisms for this outcome varied amongst the trials. Two trials used score cutoffs from different sedation scoring systems [25, 29], while two other trials reported the time spent in a predefined adequate sedation range [26, 27].

Perhaps the most important role of clonidine is as an adjunctive or sedative sparing agent. This is supported by the finding that clonidine reduces the total dose of narcotics required. This is consistent with previous studies in the perioperative setting suggesting that clonidine may be effective as an analgesic adjunct to opioids by decreasing the overall narcotic requirements [32]. The mechanism may be due to the modest anti-nociceptive effects of clonidine via stimulation of central post-synaptic alpha-2 adrenoreceptors in the spinal cord and brain stem nuclei [32]. These results support the potential role of clonidine as a narcotic-sparing sedative.

The role of clonidine as a stand-alone sedative remains unclear. In this review, only one trial used clonidine as a stand-alone sedative, compared to dexmedetomidine [27]. Less patients in the clonidine group achieved target sedation. This was largely due to concerns with hypotension, which limited the ability to increase and optimize the clonidine dose. The higher incidence of clinically significant hypotension with clonidine use is also reflected in this review. However, before definitive conclusions can be drawn, further dosing studies using variable route and delivery methods of clonidine are needed, as there is currently no standard regimen for clonidine administration. These factors may significantly impact the incidence of hypotension with clonidine use. Further, although clonidine is a significantly cheaper alternative to dexmedetomidine, dedicated cost-effectiveness analysis, taking into consideration drug efficacy, adverse effects and cost would better inform the clinician on the drugs’ practical applicability.

This systematic review has several strengths. We performed a comprehensive literature search, used the Preferred Reporting Items for Systematic Reviews and Meta-Analyses guidelines [33] and established and published a protocol [23]. Data abstraction was performed in duplicate and study authors were contacted to address missing data. Multiple clinically relevant outcomes were defined a priori and included in the analysis. Also, using GRADE methodology, we were able to report the certainty in the overall estimates of effect for our outcomes of interest.

However, there were several limitations to our analyses. There was substantial clinical heterogeneity, limiting direct comparisons between groups. The ROB was also moderately high across trials, affecting the validity of individual outcomes. These factors were accounted for in our GRADE assessments, resulting in many outcomes with low certainty in the pooled estimates. Further, the number of studies was insufficient to allow for meaningful subgroup and sensitivity analyses.

## Conclusions

Based on moderate- to low-certainty evidence, the use of clonidine did not significantly change the duration of mechanical ventilation, although it did result in a significantly decreased requirement for narcotics, however with an increased incidence of clinically significant hypotension. Until further large-scale RCTs are performed, data remains insufficient to support the routine use of clonidine as a sedative, either stand-alone or adjunctive, in the mechanically ventilated population.

## Appendix 1

The MEDLINE search strategy, including search terms and relevant Medical Subject Headings

## Appendix 2

Risk of bias assessment for each trial using the Cochrane risk of bias tool. The green symbol represents low risk of bias and the red symbol represents high risk of bias. The yellow symbol represents an unclear risk of bias.

## Appendix 3

Forest plot comparing the incidence of mortality in the intensive care unit between the clonidine group and the non-clonidine group (control). Results are depicted using a random effects model with relative risk ratio and 95% confidence intervals.

## Appendix 4

Forest plot comparing the incidence of mortality during the hospital stay between the clonidine group and the non-clonidine group (control). Results are depicted using a random effects model with relative risk ratio and 95% confidence intervals.

## Appendix 5

Forest plot comparing the duration of sedative infusions between the clonidine group and the non-clonidine group (control). Results are depicted using a random effects model with mean difference and 95% confidence intervals.

## Appendix 6

Forest plot comparing the dose of benzodiazepines used between the clonidine group and the non-clonidine group (control). Results are depicted using a random effects model with standard mean difference and 95% confidence intervals.

## Appendix 7

Forest plot comparing the level of sedation between the clonidine group and the non-clonidine group (control). Results are depicted using a random effects model with standard mean difference and 95% confidence intervals.

## Appendix 8

Forest plot comparing the incidence of withdrawal from other sedatives between the clonidine group and the non-clonidine group (control). Results are depicted using a random effects model with relative risk ratio and 95% confidence intervals.

## Appendix 9

Forest plot comparing the duration of stay in the intensive care unit between the clonidine group and the non-clonidine group (control). Results are depicted using a random effects model with mean difference and 95% confidence intervals.

## Appendix 10

Forest plot comparing the duration of stay in the hospital between the clonidine group and the non-clonidine group (control). Results are depicted using a random effects model with mean difference and 95% confidence intervals.

## Appendix 11

Forest plot comparing the incidence of clinically significant bradycardia between the clonidine group and the non-clonidine group (control). Results are depicted using a random effects model with relative risk ratio and 95% confidence intervals.

## Appendix 12

Forest plot comparing the incidence of rebound hypertension between the clonidine group and the non-clonidine group (control). Results are depicted using a random effects model with relative risk ratio and 95% confidence intervals.

## Abbreviations

ATS: American Thoracic Society
CI: Confidence interval
CINAHL: Cumulative Index to Nursing and Allied Health Literature
EMBASE: Excerpta Medica database
ESICM: European Society of Intensive Care Medicine
GRADE: Grading of Recommendations Assessment Development and Evaluation
ICU: Intensive care unit
IMV: Invasive mechanical ventilation
IV: Intravenous
MD: Mean difference
NIV: Non-invasive ventilation
RCTs: Randomized controlled trials
ROB: Risk of bias
RR: Relative risk
SCCM: Society of Critical Care Medicine
SMD: Standardized mean difference
WHO ICTRP: World Health Organization International Clinical Trials Registry Platform
